# Lifelong Cognitive Reserve, Imaging Markers of Brain Aging, and Cognitive Function in Dementia-Free Rural Older Adults: A Population-Based Study

**DOI:** 10.3233/JAD-220864

**Published:** 2023-03-07

**Authors:** Yuanjing Li, Mingqi Wang, Lin Cong, Tingting Hou, Lin Song, Xiang Wang, Lin Shi, Serhiy Dekhtyar, Yongxiang Wang, Yifeng Du, Chengxuan Qiu

**Affiliations:** a Department of Neurology, Shandong Provincial Hospital, Jinan, Shandong, P.R. China; b Aging Research Center and Center for Alzheimer Research, Department of Neurobiology, Care Sciences and Society, Karolinska Institutet and Stockholm University, Stockholm, Sweden; c Department of Imaging and Interventional Radiology, The Chinese University of Hong Kong, Hong Kong SAR, P.R. China; d Cheeloo College of Medicine, Shandong University, Jinan, Shandong, P.R. China

**Keywords:** Cognition, cognitive reserve, mild cognitive impairment, neuroimaging, old age

## Abstract

**Background:**

Cognitive reserve (CR) partly explains cognitive variability in the presence of pathological brain aging.

**Objective:**

We investigated the interplay of lifelong CR with age, sex, and brain aging markers in cognitive phenotypes among older adults with very limited education.

**Methods:**

This population-based cross-sectional study included 179 dementia-free participants (age ≥65 years; 39.7% women; 67.0% had no or elementary education) examined in 2014–2016. We assessed lacunes and volumes of hippocampus, ventricles, grey matter, white matter (WM), and white matter hyperintensities. Lifelong CR score was generated from six lifespan intellectual factors (e.g., education and social support). We used Mini-Mental State Examination (MMSE) score to assess cognition and Petersen’s criteria to define mild cognitive impairment (MCI). Data were analyzed using general linear and logistic models.

**Results:**

The association of higher lifelong CR score (range: –4.0–5.0) with higher MMSE score was stronger in women (multivariable-adjusted β-coefficient and 95% CI: 1.75;0.99–2.51) than in men (0.68;0.33–1.03) (p_interaction_ = 0.006). The association of higher CR with MCI (multivariable-adjusted odds ratio and 95% CI: 0.77;0.60–0.99) did not vary by age or sex. Among participants with low CR (<1.4[median]), greater hippocampal and WM volumes were related to higher MMSE scores with multivariable-adjusted β-coefficients being 1.77(0.41–3.13) and 0.44(0.15–0.74); the corresponding figures in those with high CR were 0.15(–0.76–1.07) and –0.17(–0.41–0.07) (p_interaction_ <0.01). There was no statistical interaction of CR with MRI markers on MCI.

**Conclusion:**

Greater lifelong CR capacity is associated with better late-life cognition among people with limited education, possibly by compensating for impact of neurodegeneration.

## INTRODUCTION

The concept of cognitive reserve (CR) has been proposed to explain the individual variability in cognitive phenotypes in the presence of pathological brain aging (e.g., reduced gray matter volume and hippocampal volume) [1, 2]. People with higher CR capacity could better cope with the brain pathologies and maintain cognitive function [3]. The CR capacity can be derived from intellectual factors experienced over the lifespan, such as early-life education attainment, adulthood socioeconomic position, marital status, occupational complexity, and mentally stimulating activity, physical exercise, and social engagement later in life [4]. Individual CR proxies (e.g., education, cognitive activity, and social engagement) have been frequently linked with greater cognitive performance and reduced risk of dementia in late life [1, 5]. In recent years, several cohort studies have investigated lifelong composite measurements of CR in association with cognitive phenotypes in late life. For example, two population-based studies have suggested that cumulative exposure to lifelong CR-enhancing factors is associated with a lower risk of mild cognitive impairment (MCI) [6] and that people with high lifelong CR capacity could maintain global cognition even in the presence of Alzheimer’s disease-related brain pathology (i.e., diffuse and neuritic plaques and neurofibrillary tangles) and gross infarcts [7]. However, most of these previous population-based studies have been conducted among highly educated urban populations, where the findings may not be generalizable to rural population or older people with no or very limited education. Individual CR proxies in rural residents are distinct from those of urban population with regard to educational attainment, lifestyle, and leisure activities. For example, rural residents are less likely to receive education or do physical activity compared to urban population [8]. In addition, educational attainment is the major contributor to lifelong CR capacity [9], considering that early-life education usually has an impact on other CR proxies over the lifespan (e.g., early-life socioeconomic status, adulthood work complexity, and late-life social engagement) [10]. Therefore, exploring the potential role of lifelong CR capacity in late-life cognitive phenotypes among rural-dwelling older adults with no or very low education might shed some light on the generalizability of CR theory.

Furthermore, the sex or gender differences in CR proxies and cognitive performance are well established, such that compared to men, women usually have lower levels of lifelong CR proxies (e.g., lower educational level and lower work complexity) [11], and poorer cognitive performance [12, 13]. There is also evidence that greater lifelong CR may be more beneficial in lowering risk of cognitive aging in women than in men, although more research is needed to understand the mechanisms [14]. In addition, the association of CR proxies with cognition varies by age, such that physical activity is related to better cognitive performance only in older people but not in younger people [15]. Accordingly, we hypothesize that the potential cognitive benefits of lifelong CR capacity vary by age and sex. This is a highly relevant issue because clarifying the possible age- and sex-variations in the association between lifelong CR capacity and cognition may help develop personalized multimodal interventions to compress cognitive morbidity.

In this population-based brain imaging study, we aimed to investigate the interplay of lifelong composite CR with pathological brain aging markers in cognitive phenotypes among rural older adults with no or very limited education while taking into account possible impact of age and sex. We hypothesized that 1) a greater lifelong CR capacity was associated with better cognitive function and the association was stronger in women than men and in older adults than younger adults; and 2) a higher lifelong CR capacity could attenuate the associations of pathological brain aging markers with MCI and poor cognitive function.

## MATERIALS AND METHODS

### Study design and participants

This was a population-based cross-sectional study. Study participants were derived from the Shandong Yanggu Study of Aging and Dementia (SYS-AD) that engaged people who were aged 60 years and older and living in the rural area of Yanlou Town, Yanggu County, western Shandong Province, China, as previously reported [12, 16]. Briefly, the baseline examination of the SYS-AD project consisted of a two-phase procedure. At phase I (a screening phase, August-December 2014), 3,277 eligible participants underwent face-to-face interviews, clinical examinations, cognitive screening tests by trained staff in the local town hospital to collect data on demographics, lifestyles, health history, and global cognitive function. During this phase, the Mini-Mental State Examination (MMSE) and the Ascertain Dementia 8-item Questionnaire (AD8) were administrated to screen for global cognitive impairment. A higher MMSE score or a lower AD8 score indicates better global cognition. At phase II (a clinical phase, December 2014-September 2015), 1,981 participants who had either MMSE score ≤24 or AD8 score ≥2 were invited to undertake full clinical and neuropsychological assessments [17, 18], and 997 (50.3% of participants who were screening positive at phase I) eventually accomplished the phase II assessments [16]. The functions of the following five cognitive domains were assessed using multiple neuropsychological tests at phase II, i.e., the Auditory Verbal Learning Test, Huashan version (short-term delayed recall, long-term delayed recall, and category-cued recall and recognition) for episodic memory; the Fuld Object Memory Evaluation, the Category Verbal Fluency test, and the Boston Naming Test for language; the Trail Making Test and Stroop interference test for executive function; the Wechsler Memory Scale-III Forward Digit Span and Backward Digit Span for attention; the Wechsler block design test and the Clock Drawing Test for visuospatial ability. The raw score of each of these tests was standardized into z-score using the age- and education-specific means and standard deviations, among participants who were free from dementia. In addition, we used the Functional Activities Questionnaire (FAQ) for assessing instrumental activities of daily living, the Hamilton Depression Rating Scale for assessing depression, and the Hachinski Ischemic Score for assessing vascular brain damage.

**Fig. 1:**
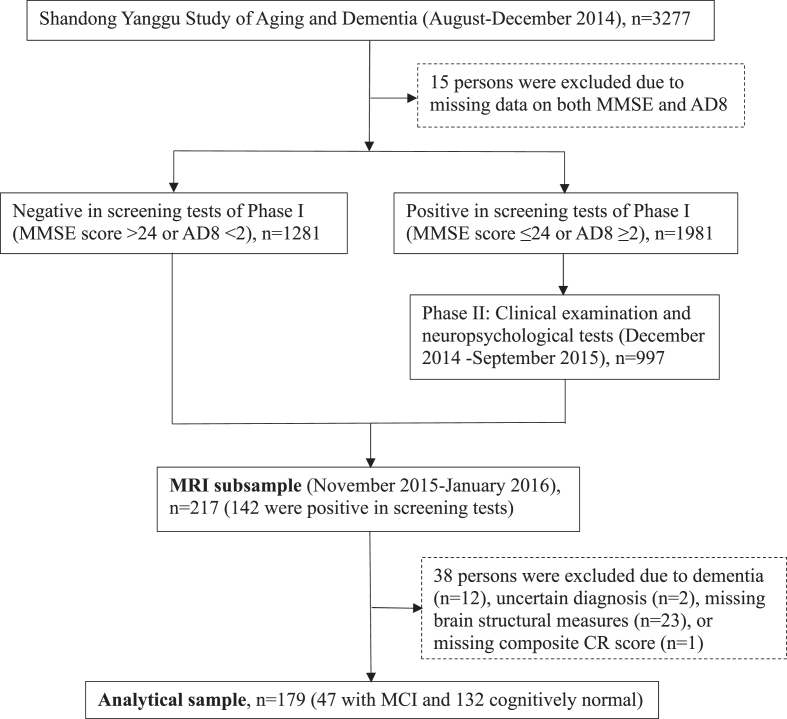
Flowchart of study participants. MMSE, Mini-Mental State Examination; AD8, Ascertain Dementia 8-item Questionnaire; CR, cognitive reserve; MCI, mild cognitive impairment; MRI, magnetic resonance imaging.

Following the clinical phase (phase II), 217 participants undertook brain magnetic resonance imaging (MRI) scans in November 2015-January 2016. Of these, 38 persons were excluded due to dementia (*n* = 12), insufficient information for the diagnosis of dementia (*n* = 2), suboptimal imaging quality, incomplete imaging sequences, or cerebral infarcts (*n* = 23) or missing composite CR score (*n* = 1), leaving 179 persons for the current analyses. Figure 1 shows the flowchart of study participants.

The protocols of SYS-AD were approved by the Ethics Committee on Human Experimentation at Shandong Provincial Hospital in Jinan, Shandong. Written informed consent was obtained from all participants, or if the participants were not able to give the consent, from the informants.

### Data collection and assessment

At phase I, we collected data through face-to-face interviews, clinical examinations, and laboratory tests. Data included demographics (e.g., age, sex, early-life education, and adulthood occupation and marital status), lifestyles (e.g., smoking, alcohol intake, and late-life physical and social activity), health conditions (e.g., hypertension, diabetes, hyperlipidemia, stroke, and coronary artery disease), and cognitive performance (e.g., MMSE score and AD8 score) [12, 16]. The Social Support Rating Scale, developed and validated among Chinese older adults, was used to assess social contact and support [19]. We categorized early-life education as no formal schooling, elementary school, and middle school or above; adulthood occupation as farmers versus non-farmers; marital status as single, divorced, or widowed versus married; late-life physical activity as less than weekly versus weekly activity; and late-life social activity into frequent versus never or occasional participation. We dichotomized late-life social support into low and high social support according to the mean score of Social Support Rating Scale [20]. We defined ever smoking and alcohol intake according to self-reported information. Hypertension was defined as self-reported history of hypertension, systolic pressure ≥140 mm Hg, or diastolic pressure ≥90 mm Hg [16]; diabetes as having a self-reported history of diabetes diagnosed by a physician or fasting serum glucose ≥7.0 mmol/L [16], and hyperlipidemia as total cholesterol level ≥6.2 mmol/L, triglyceride ≥2.3 mmol/L, low-density lipoprotein cholesterol ≥4.1 mmol/L, high-density lipoprotein cholesterol <1.0 mmol/L, or having a self-reported hyperlipidemia [16]. Stroke and coronary heart disease were ascertained according to self-reported history.

### Diagnosis of mild cognitive impairment

In SYS-AD, MCI was defined by two neurologists (L.C. and H.T.) via reviewing all records from the phases I and II examinations, following the Petersen’s criteria [12, 21]: 1) subjective cognitive concern or complaint by the subject or informant; 2) objective cognitive impairment in one or more domains beyond normal aging, according to the cognitive tests (composite z-score of any of the five cognitive domains lower than mean-1.5 standard deviation of z-scores in the population); 3) preserved global cognitive function assessed using the education-based MMSE score (MMSE score >17 for no formal schooling, >20 for elementary school, or >22 for middle school or above) [22]; 4) normal functional activities assessed by FAQ (FAQ score <6); and 5) free of dementia according to the Diagnostic and Statistical Manual of Mental Disorders, fourth edition, criteria [23].

### MRI acquisition and assessment protocol

All eligible participants were scanned at one of the three MRI centers in Liaocheng, Shandong [12]. Supplementary Table 1 provides the parameters of core MRI sequences. T1-weighted and fluid attenuated inversion recovery (FLAIR) images were processed using AccuBrain (Brainnow Medical Technology Ltd., Shenzhen, China), which is a brain quantification tool that automatically segmented brain structure and quantified global and regional brain volumes. The total intracranial volume, total grey matter (GM) volume, total white matter (WM) volume, hippocampal volume, and ventricular volume were automatically estimated using T1-weighted images following a previously in-house developed pipeline [12]. Volumes of the hippocampus, total GM, total WM, and ventricles were adjusted by total intracranial volume [24]. White matter hyperintensities (WMHs) were automatically segmented and volume of WMHs was automatically estimated using FLAIR images [12]. The WMH volume was log-transformed due to the highly skewed distribution. A trained rater (Y. L., MD) visually evaluated the number and location of lacunes using T2-weighted and FLAIR images, following the STRIVE criteria [25, 26], under the supervision of a senior neurologist (L.S.).

### Statistical analysis

In the SYS-AD total sample of participants with complete data on CR factors (*n* = 3,060), we quantified the lifelong CR capacity by generating a composite score from the structural equation model, in which early-life educational attainment, adulthood occupation and marital status, and late-life physical exercise, social support, and social activity were taken into consideration, according to previous literature (Supplementary Figure 1) [2, 4, 6, 27]. In the analytical MRI sample (*n* = 179), we used the chi-square test to compare the characteristics of participants for categorical variables and *t*-test for continuous variables by sex. We used the general linear regression model to examine the associations of lifelong CR capacity and structural brain MRI markers with MMSE score, and the logistic regression model to explore the associations of CR capacity and structural brain MRI markers with MCI. The lifelong CR capacity was treated both as continuous variable (CR score) and categorical variable (tertiles). To assess whether the associations of lifelong CR score with MMSE score and MCI varied by age and sex, we tested the statistical interactions of lifelong CR score with age (60–74 versus ≥75 years) and sex on MMSE score using the general linear regression models and the likelihood of MCI using the logistic regression models. Similarly, to evaluate whether lifelong CR levels modify the relations of brain MRI measures with cognitive outcomes, we examined the statistical interactions of lifelong CR levels with brain MRI measures on MMSE score and likelihood of MCI, in which lifelong CR score was dichotomized according to the median CR score. When a statistical interaction was detected, we further performed stratifying analysis to assess the extent and direction of the interaction. We built three statistical models in the analyses: Model 1 was adjusted for age and sex; Model 2 was additionally adjusted for smoking, alcohol intake, hypertension, hyperlipidemia, diabetes, stroke, coronary heart disease, and lifelong CR score; and in Model 3 we added, if applicable, brain MRI variables of lacunes, hippocampal volume, total GM volume, total WM volume, and WMH volume to Model 2. We applied Stata 16 Software for Windows for all the statistical analyses (StataCorp. 2019. Stata Statistical Software: Release 16. College Station, TX: StataCorp LLC.).

## RESULTS

### Characteristics of study participants

Of the 179 participants, the mean age was 69.3 years (standard deviation = 3.9), 39.7% were women, and 67.0% had no formal schooling or only attended elementary school (Table 1). Compared to men, women had lower lifelong CR score, lower total intracranial volume, and lower MMSE score, and were more likely to be farmer and have MCI, while less likely to have formal schooling, smoke, or drink alcohol (*p* < 0.01, Table 1). Men and women did not differ significantly in mean age, marital status, and other factors listed in Table 1 (*p* > 0.05).

**Table 1:** Characteristics of study participants by sex (*n* = 179)

Characteristics ^a^	Total sample, *n* = 179	Sex		
		Male, *n* = 108	Female, *n* = 71	*p*
Age (y)	69.31 (3.86)	68.98 (3.67)	69.82 (4.11)	0.157
Education, *n* (%)				<0.001
No formal schooling	42 (23.46)	7 (6.48)	35 (49.30)
Elementary school	78 (43.58)	49 (45.37)	29 (40.85)
Middle school or above	59 (32.96)	52 (48.15)	7 (9.86)
Occupation, *n* (%)				<0.001
Farmers	147 (82.12)	79 (73.15)	68 (95.77)
Non-farmers	32 (17.88)	29 (26.85)	3 (4.23)
Marital status, *n* (%)				0.762
Married	152 (84.92)	91 (84.26)	61 (85.92)
Single, divorced or widowed	27 (15.56)	17 (15.74)	10 (14.08)
Physical activity, *n* (%)				0.190
Weekly	99 (55.31)	64 (59.26)	35 (49.30)
Less than weekly	80 (44.69)	44 (40.74)	36 (50.70)
Social support level, *n* (%)				0.981
Low	83 (46.37)	50 (46.30)	33 (46.48)
High	96 (53.63)	58 (53.70)	38 (53.52)
Social activity, *n* (%)				0.595
Never or occasional	80 (44.69)	50 (46.30)	30 (42.25)
Frequent	99 (55.31)	58 (53.70)	41 (57.75)
Lifelong CR score (point)	1.28 (1.96)	1.99 (1.80)	0.21 (1.71)	<0.001
Ever smoking, *n* (%)	86 (48.04)	83 (76.85)	3 (4.23)	<0.001
Ever alcohol intake, *n* (%)	95 (53.07)	91 (84.26)	4 (5.63)	<0.001
Hypertension, *n* (%)	123 (68.72)	76 (70.37)	47 (66.20)	0.556
Hyperlipidemia, *n* (%)	33 (18.44)	19 (17.59)	14 (19.72)	0.720
Diabetes, *n* (%)	27 (15.08)	20 (18.52)	7 (39.66)	0.113
Stroke, *n* (%)	16 (8.94)	9 (8.33)	7 (9.86)	0.726
Coronary heart disease, *n* (%)	31 (17.32)	20 (18.52)	11 (15.49)	0.601
Total intracranial volume (ml)	1431.80 (149.31)	1507.55 (116.48)	1316.57 (116.77)	<0.001
Hippocampal volume (ml)	6.75 (0.73)	6.83 (0.84)	6.62 (0.50)	0.060
Ventricular volume (ml)	28.47(11.64)	29.45(12.01)	26.98 (10.96)	0.165
Total GM volume (ml)	588.91(31.48)	586.01(31.42)	593.34(31.26)	0.128
Total WM volume (ml)	485.97(29.81)	484.43(29.10)	488.30(30.91)	0.397
WMH volume (ml)	0.62 (0.69)	0.63 (0.64)	0.61 (0.77)	0.853
Lacune, *n* (%)	44 (24.58)	28 (25.93)	16 (22.54)	0.606
MMSE score, (point)	24.99 (4.78)	26.84 (3.16)	22.18 (5.45)	<0.001
MCI, *n* (%)	47 (26.26)	20 (18.52)	27 (38.03)	0.004

^a^Data were mean (standard deviation), unless otherwise specified. CR, cognitive reserve; GM, grey matter; WM, white matter; WMH, white matter hyperintensity; MMSE, Mini-Mental State Examination; MCI, mild cognitive impairment.

### Association between lifelong cognitive reserve and cognitive phenotypes

The mean lifelong CR score was 1.3 (standard deviation = 2.0; median = 1.4; range –4.0–5.0). The mean MMSE score was 25.0 (standard deviation = 4.8; median = 26; range 10–30). Out of the 179 participants, 47 (26.3%) had MCI. Each 1-point increment in the lifelong CR score was significantly associated with a higher MMSE score (β-coefficient = 0.94; 95% confidence interval [CI]: 0.60–1.28) and a lower likelihood of MCI by ∼23% after adjusting for multiple potential confounders (Table 2). In addition, compared to low tertile, the high tertile of CR score was significantly associated with a higher MMSE score and a lower likelihood of MCI (both p for linear trend <0.03), after adjusting for multiple potential confounders (Table 2).

**Table 2:** Association of lifelong cognitive reserve capacity with the Mini-Mental State Examination (MMSE) score and mild cognitive impairment (MCI) (*n* = 179)

Lifelong cognitive reserve capacity	β-coefficient (95% confidence interval), MMSE score ^a^			Odds ratio (95% confidence interval), MCI ^a^			
	Model 1	Model 2	Model 3	*n*	Model 1	Model 2	Model 3
Continuous (–4.0–5.0), per 1-point increment	0.96 (0.64–1.29)^‡^	0.97 (0.63–1.31)^‡^	0.94 (0.60–1.28)^‡^	47	0.81 (0.66–1.00)^*^	0.78 (0.63–0.97)^*^	0.77 (0.60–0.99)^*^
Categorical, tertiles
Lower (–4.0–0.5), *n* = 59	0.00 (Reference)	0.00 (Reference)	0.00 (Reference)	25	1.00 (Reference)	1.00 (Reference)	1.00 (Reference)
Medium (0.5–2.1), *n* = 60	3.72 (2.30–5.13)^‡^	3.77 (2.31–5.24)^‡^	3.76 (2.29–5.22)^‡^	14	0.49 (0.21–1.12)	0.42 (0.17–1.02)	0.46 (0.16–1.27)
Higher (2.1–5.0), *n* = 60	4.19 (2.67–5.71)^‡^	4.16 (2.60–5.73)^‡^	4.29 (2.72–5.85)^‡^	8	0.30 (0.11–0.82)^*^	0.28 (0.10–0.79)^*^	0.29 (0.09–0.90)^*^
p for trend	<0.001	<0.001	<0.001		0.015	0.012	0.029

^a^Model 1 was adjusted for age and sex; Model 2 was additionally adjusted for smoking, alcohol intake, hypertension, hyperlipidemia, diabetes, stroke, and coronary heart disease; and Model 3 was further additionally adjusted for volumes of hippocampus, ventricles, white matter hyperintensity, total grey matter, total white matter, and lacunes. ^*^p < 0.05, ^‡^*p* < 0.001.

**Fig. 2:**
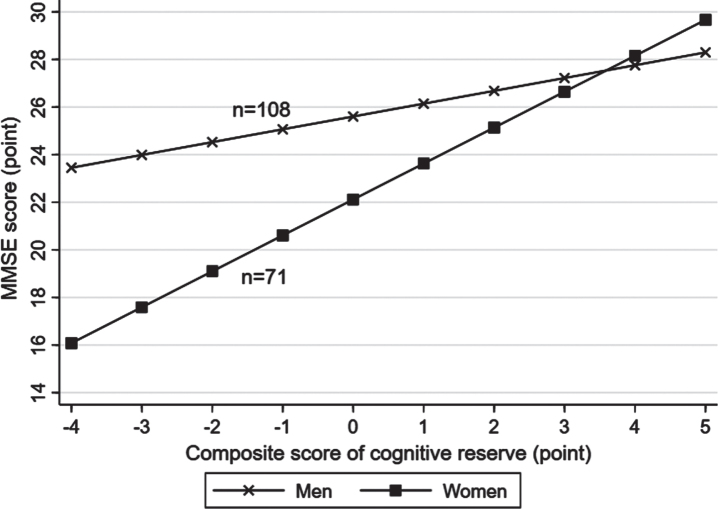
Associations of lifelong cognitive reserve with the Mini-Mental State Examination (MMSE) score by sex (*n* = 179). Models were adjusted for age, sex, smoking, alcohol intake, hypertension, hyperlipidemia, diabetes mellitus, stroke, coronary heart disease, hippocampal volume, ventricular volume, volume of white matter hyperintensity, volume of total grey matter, volume of total white matter, and lacunes.

There was a statistical interaction between the lifelong composite CR score and sex on MMSE score (p for interaction = 0.006), but not on the likelihood of MCI (p for interaction >0.05). Further analysis stratified by sex showed that the association of a higher CR score with a greater MMSE score was stronger in women (multivariable-adjusted β-coefficient = 1.75; 95% CI: 0.99–2.51) than in men (0.68; 0.33–1.03) (Fig. 2, Supplementary Figure 2). There was no statistical interaction between lifelong CR score and age groups on MMSE score or likelihood of MCI.

### Associations between brain MRI markers and cognitive phenotypes

Each 1-ml increment of hippocampal volume was significantly associated with a higher MMSE score after adjusting for age and sex (*p* < 0.05), while such an association was attenuated and became non-significant after additionally adjusting for vascular risk factors and lifelong CR capacity (Table 3). Higher hippocampal volume was marginally associated with a decreased likelihood of MCI (multivariable-adjusted OR = 0.56; 95% CI: 0.30–1.06; *p* = 0.075). Higher total WM volume was significantly associated with both a higher MMSE score and a lower likelihood of MCI, after adjusting for multiple potential confounders (*p* < 0.05, Table 3). Higher volumes of ventricles and WMH were related to an increased likelihood of MCI (*p* < 0.05), but not associated with MMSE score, after adjusting for multiple potential confounders (*p* > 0.05, Table 3). Total GM volume was not significantly associated with MMSE score or the likelihood of MCI (Table 3).

**Table 3:** Association of brain magnetic resonance imaging markers with the Mini-Mental State Examination (MMSE) score and mild cognitive impairment (MCI) (*n* = 179)

Brain magnetic resonance imaging measures	β-coefficient (95% confidence interval), MMSE score^a^			Odds ratio (95% confidence interval), MCI^a^		
	Model 1	Model 2	Model 3	Model 1	Model 2	Model 3
Hippocampal volume (3.2–8.6 ml), per 1-ml decrement	0.99 (0.13–1.84)^*^	0.63 (–0.18–1.44)	0.70 (–0.12–1.53)	0.67 (0.41–1.09)	0.70 (0.42–1.16)	0.56 (0.30–1.06)
Ventricular volume (0–73 ml), per 10-ml increment	–0.39 (–0.91–0.14)	–0.27 (–0.77–0.22)	–0.29 (–0.81–0.23)	1.60 (1.18–2.18)^†^	1.63 (1.18–2.27)^†^	1.52 (1.08–2.13)^*^
Total grey matter volume (482.3–707.4 ml), per 10-ml increment	–0.13 (–0.32–0.06)	–0.16 (–0.34–0.03)	–0.13 (–0.33–0.07)	0.94 (0.85–1.05)	0.93 (0.82–1.05)	0.93 (0.80–1.08)
Total white matter volume (378.0–592.9 ml), per 10-ml increment	0.32 (0.11–0.52)^†^	0.29 (0.09–0.48)^†^	0.26 (0.06–0.46)^*^	0.84 (0.74–0.96)^†^	0.85 (0.74–0.98)^*^	0.80 (0.68–0.95)^†^
White matter hyperintensity volume (0.2–3.9 ml), per 1-ml increment	–0.12 (–1.04–0.81)	–0.32 (–1.24–0.60)	–0.27 (–1.28–0.74)	2.54 (1.47–4.37)^‡^	2.41 (1.32–4.40)^†^	2.03 (1.05–3.95)^*^
Lacune
No (*n* = 135)	0.00 (Reference)	0.00 (Reference)	0.00 (Reference)	1.00 (Reference)	1.00 (Reference)	1.00 (Reference)
Yes (*n* = 44)	–0.51 (–1.92–0.90)	–0.19 (–1.63–1.24)	0.15 (–1.39–1.68)	2.78 (1.30–5.97)^†^	2.18 (0.91–5.21)	1.19 (0.44–3.22)

^a^Model 1 was adjusted for age and sex; Model 2 was additionally adjusted for smoking, alcohol intake, hypertension, hyperlipidemia, diabetes, stroke, coronary heart disease, and lifelong cognitive reserve score; and Model 3 was further additionally adjusted, if applicable, for volumes of hippocampus, ventricles, white matter hyperintensity, total grey matter, total white matter, and lacunes. ^*^p < 0.05, ^†^*p* < 0.01, ^‡^*p* < 0.001.

### Association of brain MRI markers with cognitive phenotypes by levels of lifelong cognitive reserve

**Fig. 3:**
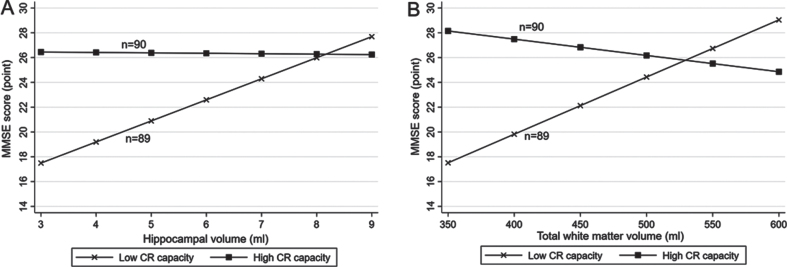
Associations of (A) hippocampal volume and (B) total white matter volume with the Mini-Mental State Examination (MMSE) score by lifelong cognitive reserve (CR) capacity (*n* = 179). A. Hippocampal volume and MMSE score by CR capacity. B. Total white matter volume and MMSE score by CR capacity. All participants were divided into two groups according to the median of lifelong CR score (median, 1.4): individuals with high CR (score ≥1.4) and those with low CR (score <1.4). Models were adjusted for age, sex, smoking, alcohol intake, hypertension, hyperlipidemia, diabetes mellitus, stroke, coronary heart disease, ventricular volume, volume of white matter hyperintensity, volume of total grey matter, and lacunes, and if applicable, for hippocampal volume and total white matter volume.

There were statistical interactions of lifelong CR levels (high versus low according to median CR score) with hippocampal volume, total GM volume, and total WM volume on MMSE score (p for interaction = 0.005, 0.016, and 0.001, respectively). Further analysis stratified by lifelong CR levels suggested that in people with a low CR level (<1.4 [median]), each 1-ml increment in hippocampal volume was significantly associated with a higher MMSE score (multivariable-adjusted β-coefficient = 1.77; 95% CI: 0.41–3.13), but there was no significant association of hippocampal volume with MMSE score in those with a high CR level (≥1.4) (multivariable -adjusted β-coefficient = 0.15; 95% CI: –0.76–1.07, Fig. 3A, Supplementary Figure 3A). Likewise, each 10-ml increment in total WM volume was significantly associated with a higher MMSE score in people with a low CR level (multivariable-adjusted β-coefficient = 0.44; 95% CI: 0.15–0.74), but not in those with a high CR level (multivariable-adjusted β-coefficient = –0.17; 95% CI: –0.41–0.07, Fig. 3B, Supplementary Figure 3B). The total GM volume was not significantly associated with MMSE score either in people with low or high CR levels (multivariable-adjusted β-coefficients = –0.18; 95% CI: –0.50–0.14 and 0.00; –0.22–0.22, respectively). There was no statistical interaction of lifelong CR levels with any of the other examined brain MRI measures on MMSE score or the likelihood of MCI.

## DISCUSSION

In this population-based study of rural-dwelling older adults with very limited education in China, we found that 1) higher lifelong CR capacity was associated with better cognitive performance (i.e., a higher MMSE score) and a lower likelihood of MCI, and the association with better cognitive performance was stronger in women than men; and 2) greater lifelong CR capacity could mitigate the associations of hippocampal volume and total WM volume with cognitive performance, such that higher hippocampal and total WM volumes were associated with better cognitive performance only in people with low CR but not in those with high CR capacity.

The association between higher lifelong CR capacity and better cognitive performance has been previously reported in the population-based studies among highly educated urban populations in Europe and USA [6, 7, 28, 29]. The Brazilian Aging Brain Study (mean years of education = 3.9) suggested that even a few years of formal education in early life could contribute to CR capacity [30]. Our findings extend the previous reports by showing that high lifelong CR capacity is associated with greater cognitive performance even among rural-dwelling older adults with no formal schooling or very limited educational attainment. Pathophysiologically, high lifelong CR capacity could improve neuroplasticity via, e.g., facilitating neurotrophic signaling, neurogenesis, and antioxidant defense in the brain, and thus maintaining brain and cognitive function [31]. In addition, higher lifelong CR capacity is linked with more efficient brain functional network and more efficient processing of information [32]. These could partly explain the association between higher lifelong CR capacity and better cognitive performance in older adults.

Notably, we found a stronger association of higher lifelong CR capacity with better cognitive performance in women than in men. The sex-disparity in the association of lifelong CR proxies or capacity with cognitive function was rarely reported in previous studies of older adults who were free of dementia. Data from European populations suggested that the association of lower levels of CR proxies (e.g., educational attainment and premorbid intelligence) with higher risk of Alzheimer’s disease was evident in women, but not in men [33], which indicated the sex-disparity in the association of CR proxies with cognitive aging. Similarly, a narrative review suggested that the association of optimal levels of CR proxies (e.g., higher educational attainment and greater occupational complexity) with lower risk of Alzheimer’s disease was stronger in women than in men [14]. Evidence from the population-based studies that investigate the sex-specific association of lifespan composite CR capacity with cognitive phenotypes of brain aging is still lacking. The possible mechanisms underlying the sex differences in CR-cognitive phenotype association are unclear. It is worth noting that in rural China, compared with men, women usually have an unequal access to education, and accordingly, would result in the relatively poorer lifelong CR proxies [11]. The relationship of greater lifelong CR with better cognitive performance is particularly evident in women even though women had a lower lifelong CR capacity than men. This highlights the importance of lifespan CR in late-life cognitive health, even among women in the low- and middle-income countries who were living in rural communities and had received no or very limited education. This has implication for the development of precision interventions, and thus, for achieving the goal of delaying the onset of dementia [34].

The associations of structural brain aging markers (e.g., larger WMH volume, lower WM volume, and enlarged ventricles) with MCI found in our study were in agreement with reports from previous cohort studies of older adults [35, 36]. Neuronal loss in old age is accompanied by cognitive deterioration and development of MCI [37], which could explain the observed associations of reduced white matter and enlarged ventricles with an increased likelihood of MCI. The association of WMHs with MCI is largely due to neuronal demyelination and damage of cerebral small vessels, although the cognitive influences of WMH burden may be less prominent than that of brain atrophy or reduced brain tissue volume [38, 39]. In addition, we found that the presence of lacunes was associated with a nearly 3-fold increased likelihood of MCI, but the association was largely attributable to other markers of cerebral small vessel disease (e.g., WMH), which was in line with the previous report from the Rotterdam study [40].

Our study showed no evidence for the potential modifying role of lifelong CR capacity in the relationship of cerebral microvascular lesions (i.e., lacunes and WMHs) with cognitive performance, which was consistent with the previous reports [41, 42]. Instead, we found that high CR capacity could mitigate the associations of lower hippocampal and total WM volumes with poorer global cognition. Neuropathological and neuroimaging studies revealed that hippocampal atrophy partly reflected AD-related pathology and hippocampal sclerosis [43, 44], and that reduced hippocampal and total WM volumes could reflect the neurodegenerative processes in brain aging [45, 46]. Therefore, our finding highlights the compensatory effect of lifelong high CR capacity on cognitive consequences of neurodegenerative lesions. In line with this finding, the Korean brain aging study found that favorable CR proxies (e.g., high education and intellectually stimulating occupation) could counteract the cognitive consequence of neurodegenerative pathology (i.e., amyloid-β deposition) [47]. Likewise, data from neuropathological studies also suggested that high lifespan CR capacity could mitigate the detrimental effect of neurodegenerative pathology (i.e., amyloid-β and tau tangle pathology) on cognitive performance [48, 49]. Compared to people with lower CR, those with higher CR capacity exhibit more functionally active compensatory network in healthy brain tissues to maintain cognitive performance, when brain pathology occurs [50]. This might partly explain how high CR capacity could help maintain cognitive ability in the presence of neurodegenerative lesions. Nevertheless, more prospective cohort studies are needed to better understand the mechanisms underlying the potential differential roles of lifespan CR capacity in compensating cognitive consequences of cerebral microvascular and neurodegenerative pathologies in older adults.

The major strength of this study refers to the population-based design that targeted rural older adults with very limited education, in which lifelong composite CR capacity was generated from multiple CR proxies over the lifespan and cognitive data were integrated with high-quality structural brain MRI data. However, our study also has limitations. First, as a cross-sectional study, we were not able to determine the causal relationship between lifelong CR capacity and cognitive outcomes, and the observed cross-sectional associations were subject to selective survival bias that might usually lead to the underestimation of the true association. Second, relevant biomarkers for brain aging (e.g., amyloid-β and tau proteins) in central nervous system were not available in our study. Third, due to relatively small sample size, the statistical power might be limited for detecting a weak or moderately strong association between exposures and outcomes. Finally, participants in the MRI sample were relatively younger and more likely to be men compared to the whole SYS-AD sample [12], which should be kept in mind when generalizing our finding to the other populations.

In summary, this population-based MRI study indicates that among rural Chinese older adults with no or very limited education, high lifelong CR capacity is associated with better cognitive performance and that the association is stronger in women than in men. In addition, high CR capacity could mitigate the associations of lower hippocampal and total WM volumes with worse cognitive performance, supporting the compensatory effect of high CR capacity on cognitive consequences of neurodegenerative pathology. These findings highlight the importance of the lifelong cumulative CR capacity in maintaining cognitive health in late life, even among older adults with very low education.

## Supplementary Material

Supplementary Material

## Data Availability

Data on which this study is based are derived from the population-based SYS-AD project. Access to these anonymized SYS-AD data will be available upon reasonable request and approval by the Department of Neurology, Shandong Provincial Hospital, Shandong, P. R. China.
