# Supplementary material for: Lifelong Cognitive Reserve, Imaging Markers of Brain Aging, and Cognitive Function in Dementia-Free Rural Older Adults: A Population-Based Study
Source: J Alzheimers Dis. 2023 Mar 7;92(1):261–72. doi: 10.3233/JAD-220864 (PMC10041437; doi:10.3233/JAD-220864)
Supplement: Supplementary Material [file jad-92-jad220864-s001.pdf]

Supplementary Material

Lifelong Cognitive Reserve, Imaging Markers of Brain Aging, and Cognitive Function in Dementia-Free Rural Older Adults: A Population-Based Study

Supplementary Table 1. Parameters of core magnetic resonance imaging sequences in the three scan centers

MRI sequences	Repetition time (ms)	Echo time (ms)	Spatial resolution (mm^3)
**Liaocheng Third People's Hospital (GE Healthcare-Signa 1.5T, USA)**			
Sagittal 3D sT1W	10.12	3.25	0.60 × 0.60 × 0.60
Axial T2W	3000.00	128.50	0.47 × 0.47 × 5.00
Axial FLAIR	8002.00	158.10	0.47 × 0.47 × 5.00
**Liaocheng People's Hospital (Philips-Achieva 3.0T, The Netherlands)**			
Sagittal 3D sT1W	6.90	3.20	1.10 × 1.10 × 1.10
Axial T2W	2596.80	80.00	0.45 × 0.45 × 6.00
Axial FLAIR	7000.00	120.00	0.45 × 0.45 × 6.00
**Liaocheng Brain Hospital (Siemens-Magnetom Avanto 1.5T, Germany)**			
Sagittal 3D sT1W	2400.00	3.60	1.30 × 1.30 × 1.20
Axial T2W	4500.00	89.00	0.72 × 0.72 × 7.15
Axial FLAIR	8000.00	106.00	0.90 × 0.90 × 7.15

3D sT1W, three-dimentional standardized T1-weighted; T2W, T2-weighted; FLAIR, fluid attenuated inversion recovery

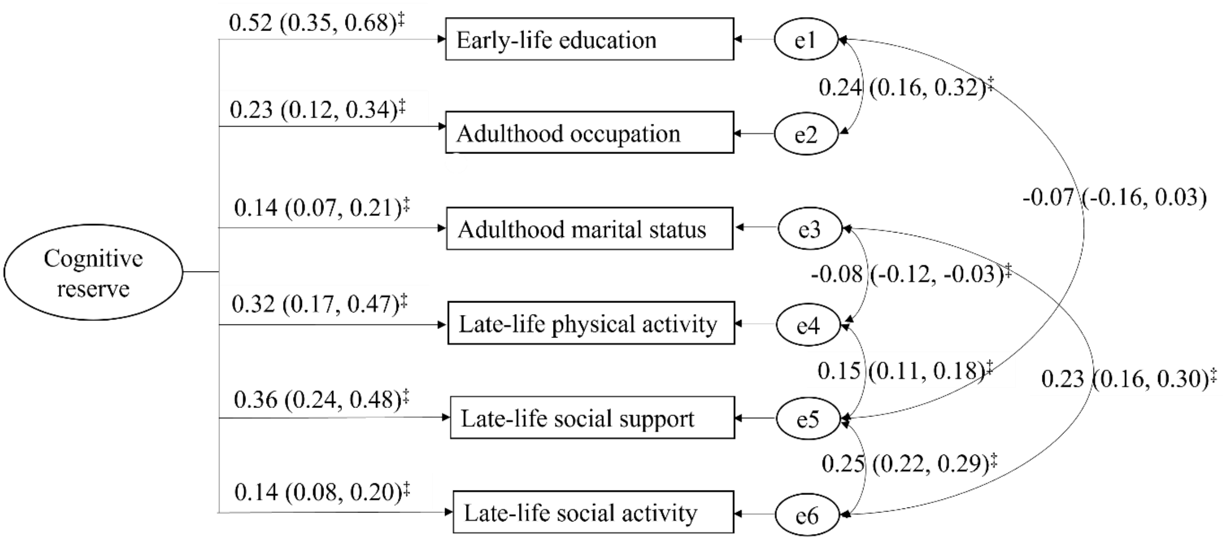

Supplementary Figure 1. Standardized estimates for the lifelong composite cognitive reserve score derived from the total sample (n=3,060). The values indicate the β-coefficients (95% confidence intervals) of the six observable factors over the life course used to generate the lifelong composite cognitive reserve score from the structural equation models. e1, e2, e3, e4, e5, and e6 represent the measurement error for each of the six observable factors in estimating the composite cognitive reserve score. Fit statistics of the structural equation model: χ^2=7.37, p=0.061; Comparative fit index=0.996; Standardized root mean squared residual=0.010; Root mean squared error of approximation=0.022; Modification index: 6.57.

‡p<0.001.

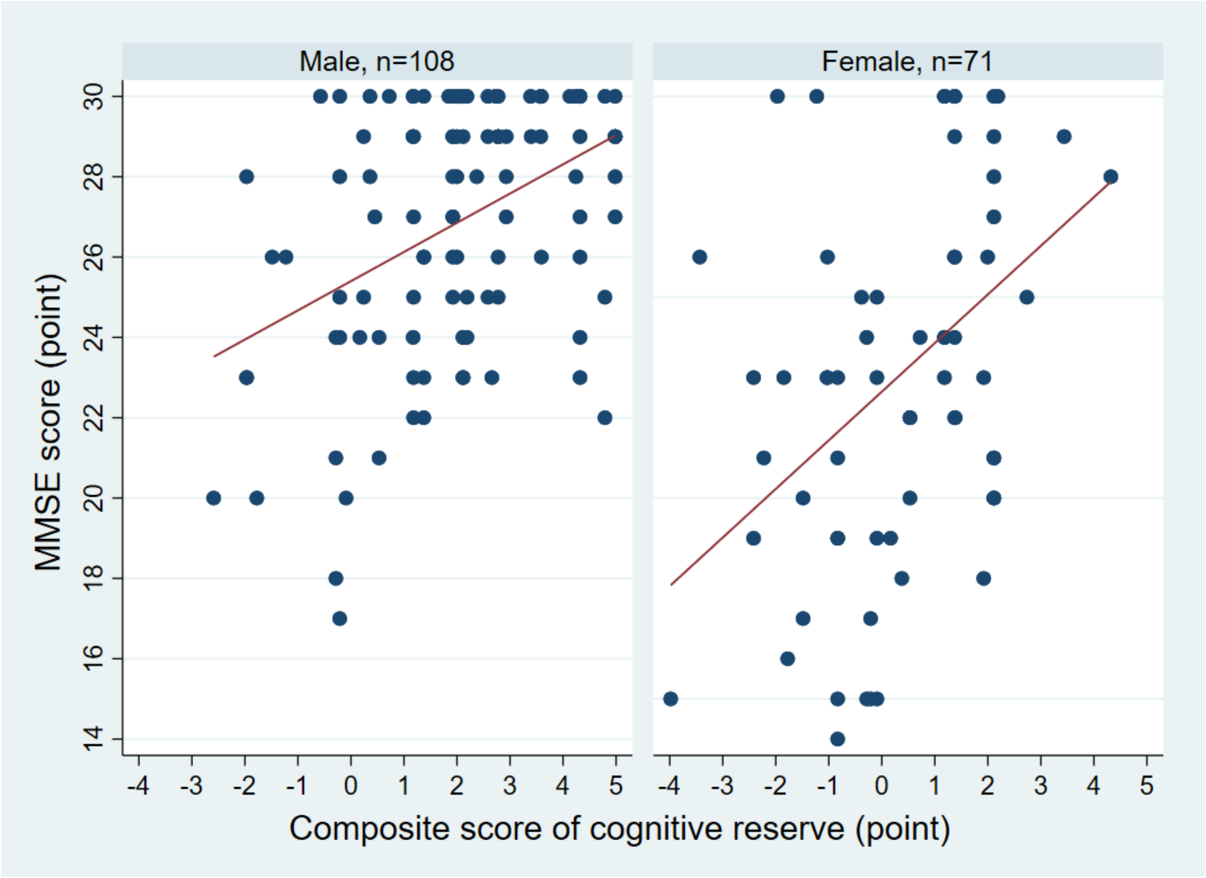

Supplementary Figure 2. Associations of lifelong cognitive reserve with the Mini-Mental State Examination (MMSE) score by sex, in two-way graph (n=179).

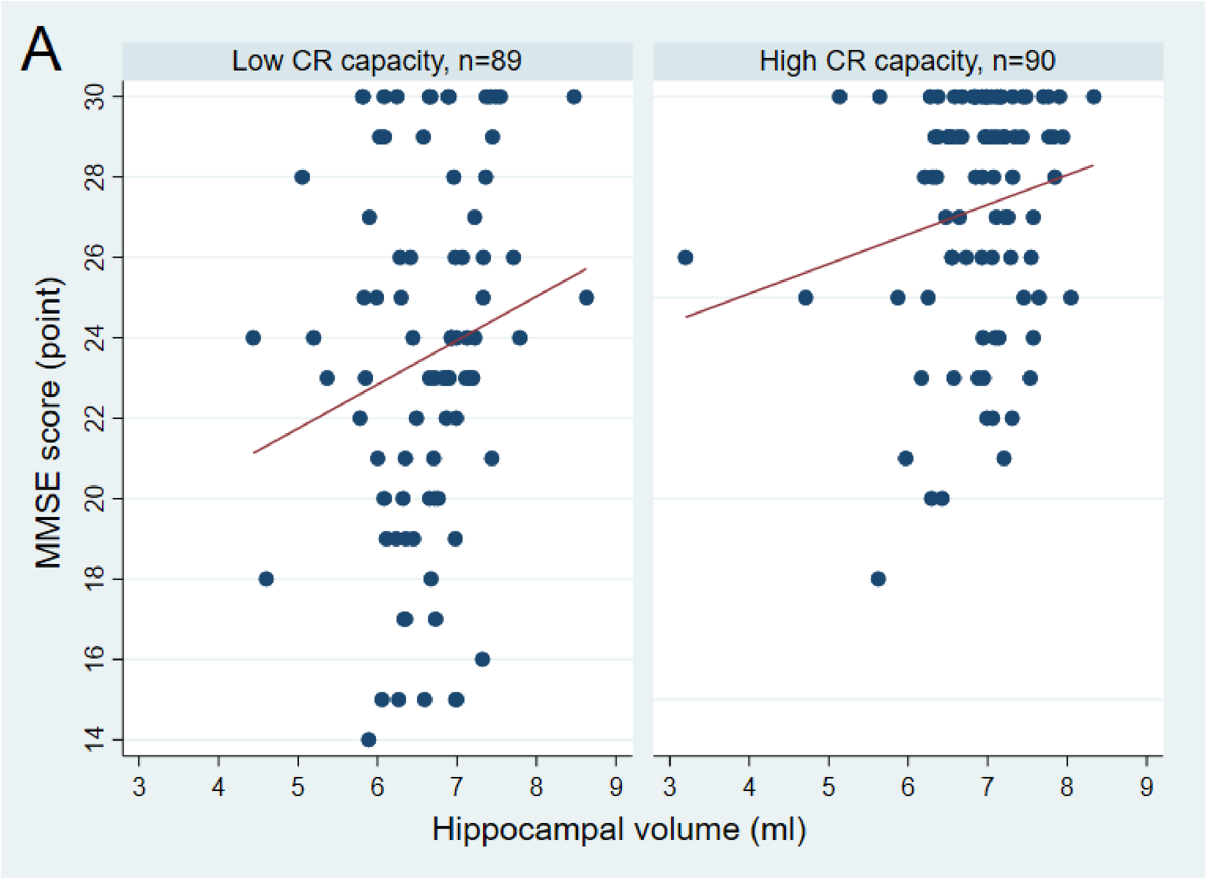
A
Low CR capacity, n=89
High CR capacity, n=90
MMSE score (point)
30
28
26
24
22
20
18
16
14
3
4
5
6
7
8
9
Hippocampal volume (ml)

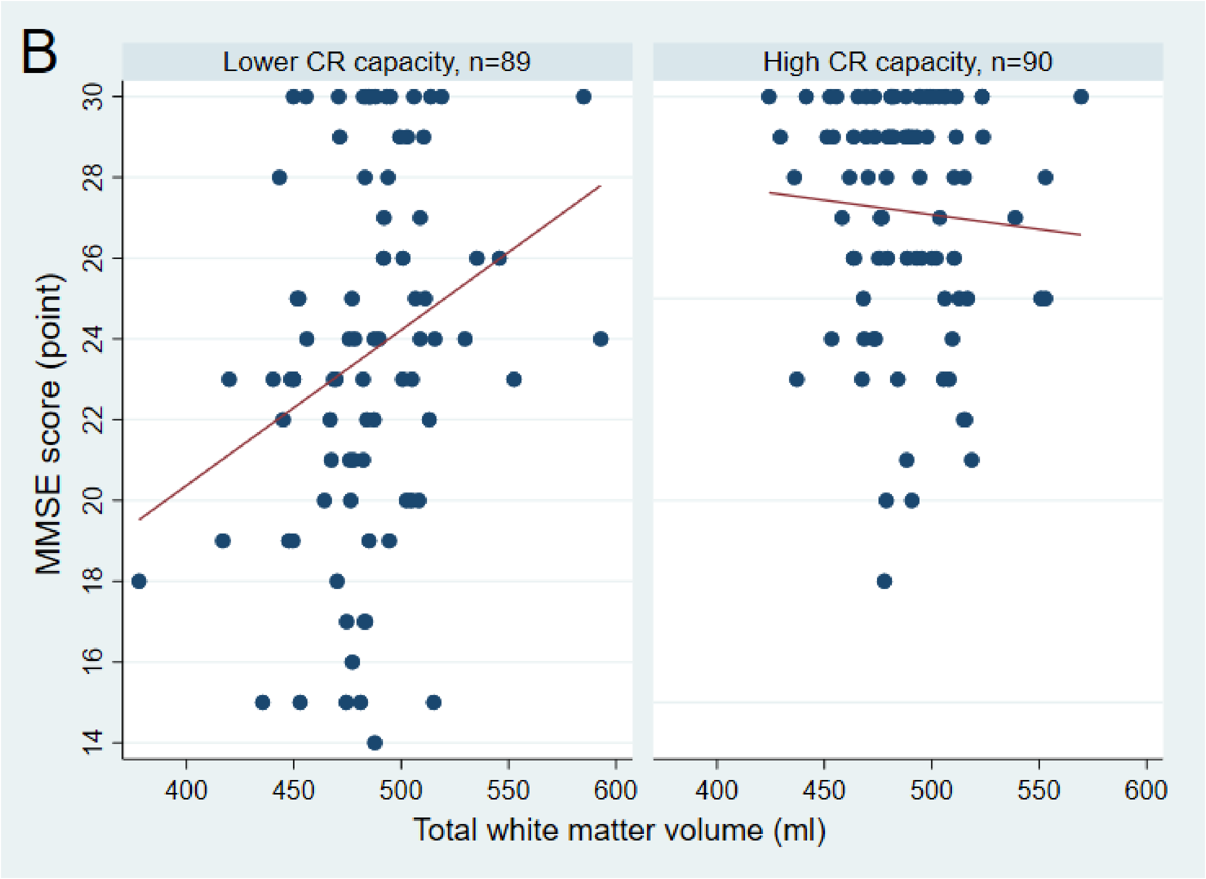

Supplementary Figure 3. Associations of (A) hippocampal volume and (B) total white matter volume with the Mini-Mental State Examination (MMSE) score by lifelong cognitive reserve (CR) levels, in two-way graph (n=179). A. Hippocampal volume and MMSE score by CR capacity. B. Total white matter volume and MMSE score by CR capacity.
